# Cortical markers of auditory stream segregation revealed for streaming based on tonotopy but not pitch

**DOI:** 10.1121/1.5065392

**Published:** 2018-10-29

**Authors:** Dorea R. Ruggles, Alexis N. Tausend, Shihab A. Shamma, Andrew J. Oxenham

**Affiliations:** Department of Psychology, University of Minnesota, 75 East River Parkway, Minneapolis, Minnesota 55455, USA; Electrical and Computer Engineering Department & Institute for Systems, University of Maryland, College Park, Maryland 20740, USA; Department of Psychology, University of Minnesota, 75 East River Parkway, Minneapolis, Minnesota 55455, USA

## Abstract

The brain decomposes mixtures of sounds, such as competing talkers, into perceptual streams that can be attended to individually. Attention can enhance the cortical representation of streams, but it is unknown what acoustic features the enhancement reflects, or where in the auditory pathways attentional enhancement is first observed. Here, behavioral measures of streaming were combined with simultaneous low- and high-frequency envelope-following responses (EFR) that are thought to originate primarily from cortical and subcortical regions, respectively. Repeating triplets of harmonic complex tones were presented with alternating fundamental frequencies. The tones were filtered to contain either low-numbered spectrally resolved harmonics, or only high-numbered unresolved harmonics. The behavioral results confirmed that segregation can be based on either tonotopic or pitch cues. The EFR results revealed no effects of streaming or attention on subcortical responses. Cortical responses revealed attentional enhancement under conditions of streaming, but only when tonotopic cues were available, not when streaming was based only on pitch cues. The results suggest that the attentional modulation of phase-locked responses is dominated by tonotopically tuned cortical neurons that are insensitive to pitch or periodicity cues.

## I. INTRODUCTION

Biologically important sounds, including speech, are rarely presented in isolation, but instead form mixtures with other sounds in the acoustic environment. The auditory system must therefore segregate the elements of a target sound from the complex background, and fuse those elements together into a perceptual stream that can be followed over time. Following from early studies on cortical correlates of attention (Hillyard *et al.*, 1973), recent human studies have revealed strong attentional modulation effects on auditory streams, with responses to an attended target stream enhanced and/or the background suppressed, both for non-speech stimuli (e.g., Gutschalk *et al.*, 2008; Elhilali *et al.*, 2009; Xiang *et al.*, 2010) and speech (e.g., Mesgarani and Chang, 2012; Zion Golumbic *et al.*, 2013; O'Sullivan *et al.*, 2015). Although the effects are clear, uncertainty remains regarding the underlying neural mechanisms that facilitate the streaming and attentional modulation of speech (Mesgarani *et al.*, 2014). Some studies have suggested early influences of attention, extending down to the brainstem (Lukas, 1981) and even the cochlea (Maison *et al.*, 2001), but most studies have concluded that attentional modulation is not evident prior to auditory cortex. Indeed, the earliest cortical responses, with latencies of 20–30 ms, responding to modulation rates around 40 Hz, also show little evidence of attentional modulation (e.g., Gutschalk *et al.*, 2008).

One important acoustic feature that allows for perceptual segregation is the spectral content of sounds. Segregation based on differences in spectral content occurs via the frequency-to-place mapping, or tonotopic organization, that is established in the cochlea and is maintained throughout the auditory pathways up to and including the auditory cortex. Perceptual studies have shown that stream segregation can occur on the basis of tonotopic separation between alternating sounds (Miller and Heise, 1950; van Noorden, 1977; Hartmann and Johnson, 1991), and some physiological studies have reported correlates of perceptual streaming of pure-tone sequences, even in the absence of physical stimulus changes, in both subcortical (Yamagishi *et al.*, 2016) and cortical (Gutschalk *et al.*, 2005) responses.

Perceptual studies have also shown that segregation can be based on higher-level features, such as pitch, timbre, or perceived location (Vliegen and Oxenham, 1999; Roberts *et al.*, 2002; David *et al.*, 2015; Javier *et al.*, 2016) and combinations thereof (Tougas and Bregman, 1985; Woods and McDermott, 2015). Attention may enhance the responses of spectrally tuned neurons that form the basis of the tonotopic representations within human auditory cortex (e.g., Formisano *et al.*, 2003; Moerel *et al.*, 2012), but may also enhance the responses of neurons that respond to higher-level features, such as pitch (Penagos *et al.*, 2004; Bendor and Wang, 2005; Norman-Haignere *et al.*, 2013; Allen *et al.*, 2017). Because different talkers differ on multiple dimensions, it is not possible to determine whether the reported cortical correlates of attention to speech (Mesgarani and Chang, 2012) are based on tonotopic cues, other higher-level features, or a combination of both. A recent study presented one talker to each ear, providing complete peripheral separation between the male talkers (O'Sullivan *et al.*, 2015). However, in more natural situations, the signal from each talker will be present at both ears, rendering it less clear how neural segregation might occur.

**FIG. 1.**
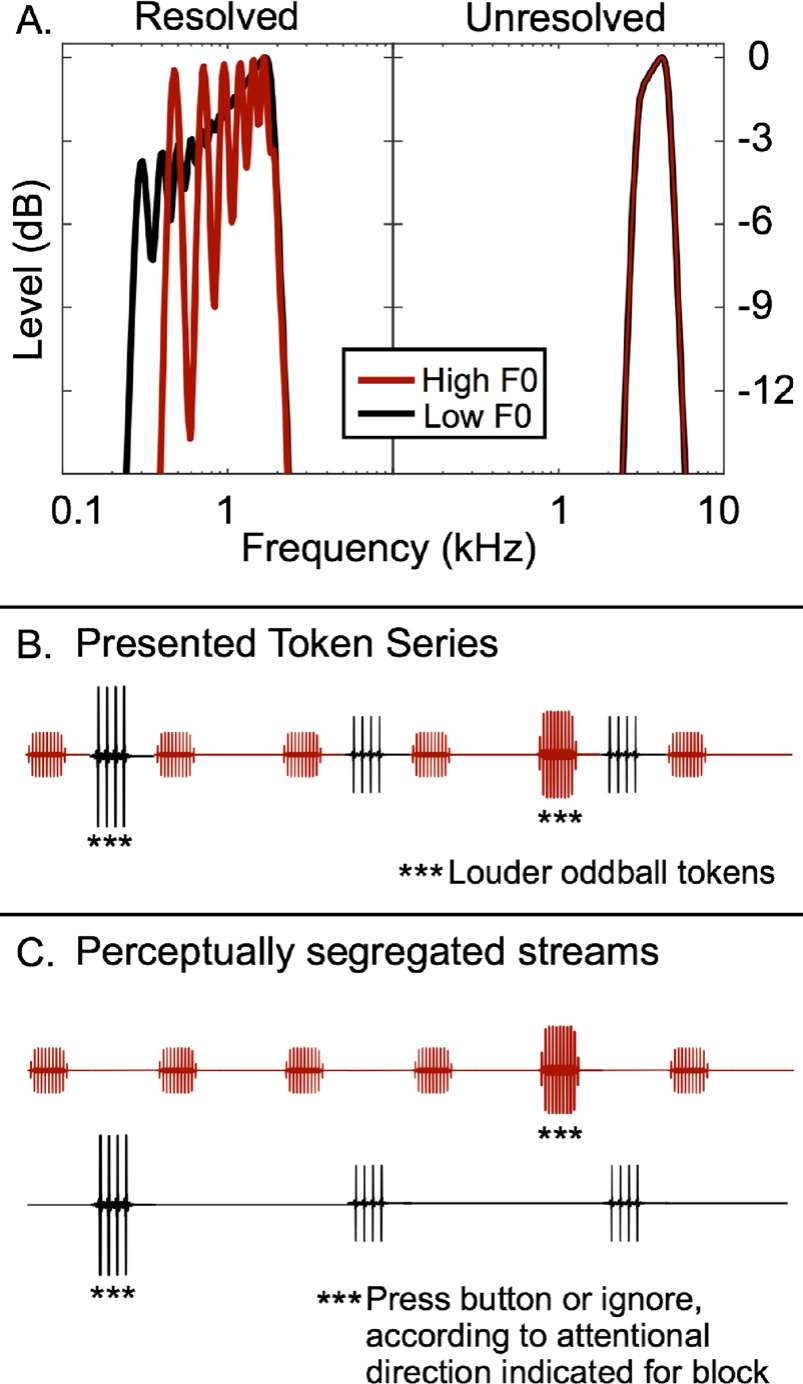
(Color online) Schematic diagram of the stimuli used in this experiment. (A) Excitation pattern representation of the tones using a model of effective auditory responses by Glasberg and Moore (1990), showing that the tones in the low spectral condition produced individual peaks in the excitation pattern and so can be considered spectrally resolved, whereas the tones in the high spectral condition did not produce clear individual spectral peaks and so can be considered spectrally unresolved. (B) Schematic diagram of the tone sequence, as presented. The higher-*F*0 A tones (red) are interleaved with the B tones (black) to form a pattern of repeating triplets. Certain tones, selected at random, are increased in level by 6 dB to produce oddballs. (C) Schematic diagram of the tone sequence, as perceived when the A and B tones form separate perceptual streams. The task of the participants was to attend to just one of the streams and report oddballs in only that stream.

The present study had two main aims. The first aim was to determine whether selective attention enhances the neural correlates of streams that are defined in terms of differences in either spectral differences and pitch, or just pitch. The second aim was to test for the existence of subcortical neural correlates of streaming and/or attention. Sequences of harmonic complex tones that alternated in fundamental frequency (*F*0) were either filtered to contain low-numbered, spectrally resolved harmonics or filtered to contain only high-numbered, spectrally unresolved harmonics. Resolved harmonics can be heard out as individual tones (Plomp, 1964; Bernstein and Oxenham, 2003), and have been shown to produce separate peaks of neural activity corresponding to each harmonic in other species (e.g., Cedolin and Delgutte, 2005; Fishman *et al*., 2013); unresolved harmonics cannot be heard out, and do not produce individual peaks of activity, meaning that changes in *F*0 do not produce changes in the overall pattern of activity along the tonotopic axis (Houtsma and Smurzynski, 1990; Shackleton and Carlyon, 1994; Cedolin and Delgutte, 2005; Fishman *et al*., 2013); see Fig. 1. A behavioral task was employed to encourage the perceptual segregation of the alternating tone sequences and to focus attention on one of the two streams. Recordings using electroencephalography (EEG) were made that measured both high- and low-frequency envelope-following responses (EFR), reflecting primarily subcortical and cortical neural populations, respectively, to identify potential correlates of streaming and attention.

The results revealed cortical modulation that reflects streaming and attention, but only in conditions that provided tonotopic differences between the tones; in conditions with only unresolved harmonics, and therefore no tonotopic differences, no significant modulation of cortical responses was observed. In addition, no brainstem or midbrain correlates of streaming or attention were observed in any condition.

## II. METHODS

### A. Participants

Twenty-eight (15 female, 13 male) young adult listeners [mean age 21.7 years; standard deviation (SD) = 3.6 years] were recruited from the University of Minnesota community. All participants had pure-tone hearing thresholds of 20 dB hearing level (HL) or better in both ears at octave frequencies between 250 Hz and 8 kHz. All participants provided written informed consent, and the protocol was approved by the Institutional Review Board of the University of Minnesota. All participants also completed a short screening protocol, described below, to ensure that they were able to perform the behavioral task. Nineteen participants (nine female, ten male) passed the screening task and completed the entire experiment. The participants who completed the experiment were aged between 18 and 32 years of age (mean = 21.9 years) and reported between 0 and 14 years of musical training (mean = 5.3 years).

### B. Stimuli

The stimuli were 1-min blocks of alternating harmonic complex tones, presented in a repeating ABA-ABA- triplet sequence, where the A tone was higher in *F*0 than the B tone. Each tone was 50 ms long, including 10-ms raised-cosine onset and offset ramps. Tones within a triplet were separated by 25-ms gaps, and each triplet was separated by a gap of 100 ms (as if every second B tone was silenced within an alternating ABABABA sequence). The repetition rate of the higher A tones was therefore 6.67 Hz, and the repetition rate of the lower B tones (and the entire triplet pattern) was 3.33 Hz. Each harmonic complex tone was generated in sine phase and bandpass filtered into a fixed spectral region. The *F*0 of the B tone was always 100 Hz and the *F*0 of the A tone was either 1 semitone higher (106 Hz) or 15 semitones higher (238 Hz). With a 1-semitone *F*0 difference, termed the small-separation condition, the two tones were likely to form a single perceptual stream, eliciting the percept of a galloping rhythm; with a 15-semitone *F*0 difference, termed the large-separation condition, the two tones were most likely to form two separate perceptual streams of isochronous tones (Vliegen and Oxenham, 1999). Control conditions were also tested, in which only the A tones (at 238 Hz), or only the B tones (at 100 Hz) were presented; these were termed the single-stream conditions.

The stimuli were filtered into either a low spectral region (300–1800 Hz) so that the complexes contained some spectrally resolved harmonics, or a high spectral region (3000–4500 Hz) so that no harmonics were spectrally resolved (the lowest harmonic number within the pass band was always greater than 12); see Fig. 1.

The tones were presented at an overall root-mean-square (rms) level of 70 dB sound pressure level (SPL) in either positive or negative polarity, and were embedded in spectrally notched threshold-equalizing noise (TEN) at 50 dB SPL per ERB, to reduce potential off-frequency listening and audible distortion products (Moore *et al.*, 2000; Oxenham *et al.*, 2009). The TEN was generated from 50 Hz to 6 kHz with a spectral notch between 250 Hz and 2 kHz for the resolved-harmonics conditions and a spectral notch between 2.7 and 5.25 kHz for the unresolved-harmonics conditions. The noise was generated as a 1-s circular token, repeated to create a 70-s source sound. For each block, 61 s of noise was randomly sampled and added to the triplet stimuli with a 1-s onset lead. Level oddballs in both the A- and B-tone sequences were used to monitor stream segregation and attention. Each sequence (A and B tones) contained six oddball tokens per block that were presented at a level 6 dB higher than the regular tokens. Oddballs were prevented from occurring in the first or last triplets of the sequence, and were restricted from occurring within the same triplet in both the A- and B-tone sequences and from being separated by fewer than two regular tokens within a given sequence.

### C. Procedure

The experiment was carried out in a sound-attenuating and electrically shielded booth. The participants were seated comfortably with a number pad on their laps and were instructed to attend selectively to either the high or low tones within each 60-s block and to press the button on their key pad every time an oddball occurred in the attended tones. They were seated in clear view of a computer monitor, which indicated the target stream for the current block (high or low) and provided an immediate acknowledgment of each button press. However, no correct-answer feedback was provided. The participants were required to respond to the oddball tokens within the attended stream by pressing a button immediately after each detected oddball, and to suppress responses to oddballs in the unattended streams.

The task proved difficult for some individuals, even with a 15-semitone separation between high- and low-*F*0 tokens, so a training/screening protocol was introduced to ensure that each listener could perform the task adequately prior to data collection. The first two blocks consisted of dichotic stimuli: the high-*F*0 (A) tones were presented to the right ear (attended in the first block), and the low-*F*0 (B) tones were presented to the left ear (attended in the second block). The results and strategies in the two dichotic blocks were discussed with each participant before they moved on to four blocks of diotically presented stimuli with the large *F*0 difference. Participants completed two blocks with resolved harmonics (attend high and attend low) and two blocks with unresolved harmonics (attend high and attend low), and were provided with feedback. In order to pass a set of blocks, the participants were required to accurately respond to at least four of the six oddballs in the attended stream and to respond to two or fewer of the oddballs in the unattended stream in all four blocks. The participants were required to pass two consecutive sets of these four blocks in order to be included in the experiment. Unlimited attempts at the four-block sets were allowed. The participants who failed to meet this criterion often attempted it over ten times before discontinuing the experiment and most commonly failed the attend-low condition with unresolved harmonics. Those who met the criteria for the study generally did so within about four sets. They then completed a set of eight blocks that included both large- and small-*F*0 separation conditions with both resolved and unresolved harmonics before undertaking the three EEG test sessions.

During the sessions with EEG, simultaneous low- and high-frequency EFRs were recorded to the 1-min-long presentations of the stimuli. The low-frequency EFRs corresponded to the presentation rates of the A and B tones (6.67 and 3.33 Hz, respectively) and the individual tones (13.3 Hz, including the “missing” B tone after each triplet), reflecting cortical activity. The high-frequency EFRs corresponded to the *F*0s of the tones (between 100 and 238 Hz) and are thought to reflect primarily subcortical activity (Bidelman, 2018). The stimuli were generated using matlab (The Mathworks, Natick, MA) and were played to participants via a Tucker Davis Technologies (Alachua, FL) real-time processor with headphone buffer and ER1 insert earphones (Etymotic Research, Elk Grove Village, IL). The EEG measurements were acquired using a BioSemi (Amsterdam, Netherlands) active electrode system with a sampling rate of 4096 Hz and 32 channels, referenced to averaged mastoid electrodes. Two blocks of each of the 24 conditions (resolved or unresolved harmonics; small or large *F*0 difference or single sequence; attend high or low; positive or negative stimulus polarity) were tested in each of the three sessions, resulting in 48 blocks per session, presented in random order. The participants were allowed to self-initiate each block, allowing for rest as needed between blocks. All participants completed each EEG session in 90 min or less.

### D. EEG analysis

Finite impulse response (FIR) filters with linear phase response using 800 taps were applied to the EEG recordings to separate low-frequency (0.5–30 Hz) from higher-frequency (70–1000 Hz) responses. Data from the low-frequency band were first epoched into 1-min events corresponding to presentation blocks. The 1-min EEG blocks were re-referenced to averaged mastoid electrodes and baselined to the 25-ms period prior to stimulus onset. The re-referenced and baselined 1-min blocks were further segmented into eight-triplet events with 50% (four triplets) overlap, resulting in 96 epochs of 2.4 s, each baselined to the preceding triplet (300 ms). Combining data from three sessions resulted in 288 events in positive polarity and 288 in negative polarity for each experimental condition.

Data from the high-frequency band were also epoched into 1-min blocks, re-referenced, and baselined before responses to the single 50-ms tokens were isolated. Individual tokens were baselined to the mean of the 75 ms preceding the token. Each test session produced 796 high-*F*0 tokens and 398 low-*F*0 tokens for each block type, and combining data from the three sessions resulted in 2388 high-*F*0 tokens in each polarity and 1194 low-*F*0 tokens in each polarity for each experimental condition.

The EEG signal in the 70–1000-Hz band sometimes contained an unexpected artifact at harmonics of 50 Hz, with increasing magnitude with harmonic number, even in the absence of 100 Hz stimuli. Given the significance of 100 Hz as an experimental frequency, a denoising source separation (DSS) routine was applied to reduce the role of any artifact in the analysis. Using the NoiseTools matlab toolbox, the epoched data (time × channel × trial) were orthogonalized through principal component analysis (PCA), the components were normalized, and the data were rotated and projected on the orthogonalized reference axes to remove the first two components (time-shift PCA) (Särelä and Valpola, 2005; de Cheveigne and Simon, 2007, 2008). The resultant data no longer exhibited visible artifacts at multiples of 50 Hz.

**FIG. 2.**
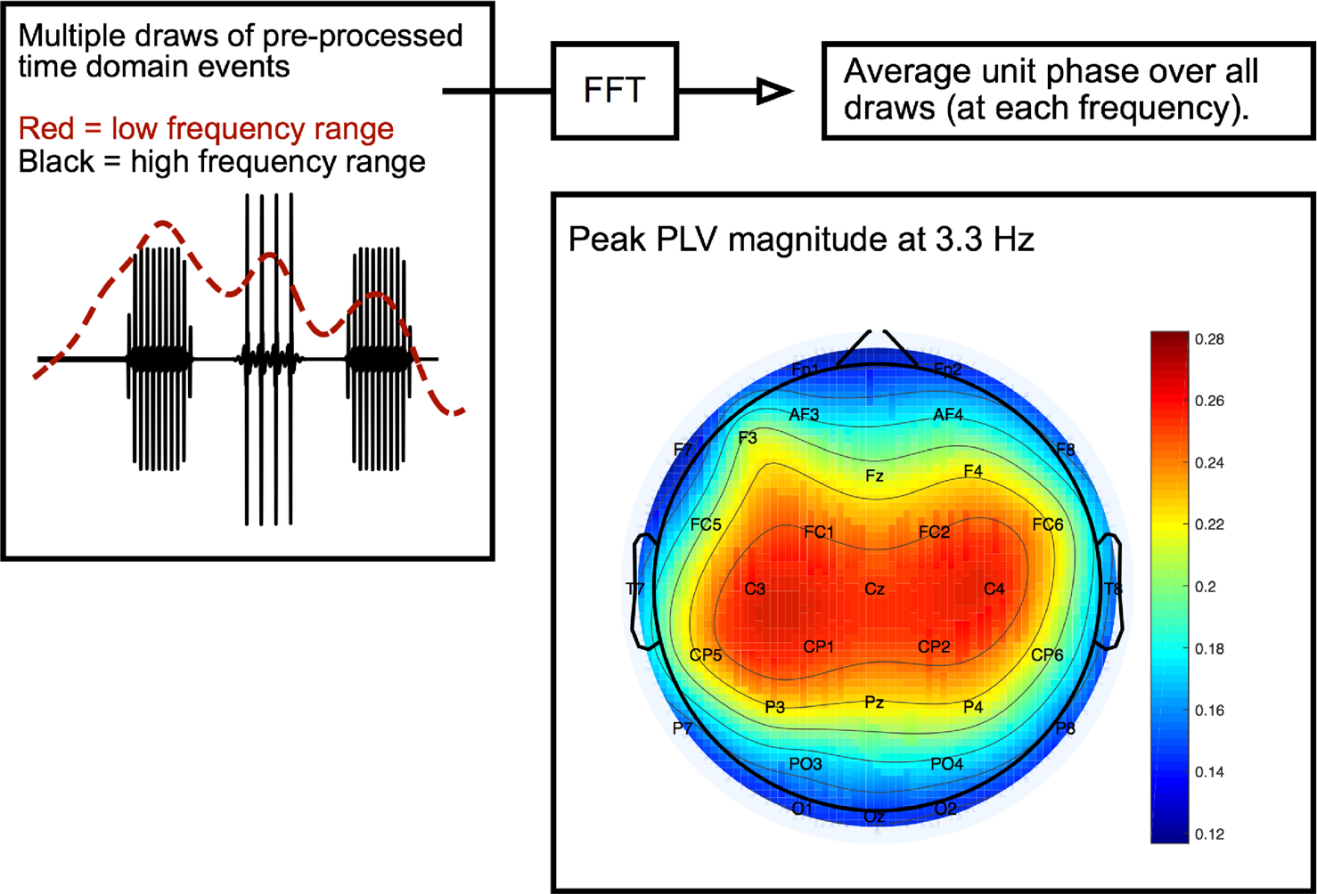
(Color online) Schematic diagram of the neural envelope-following responses to the low- and high-frequency components within the EEG signal (red and black lines, respectively). The heat map shows the distribution across the scalp of the amplitude of the peak EFR component at the low EFR frequency of 3.3 Hz.

The envelope phase-locking value (PLV; Tallon-Baudry *et al.*, 1996; Ruggles *et al.*, 2012) was computed for all electrodes, frequencies, and conditions. The PLV is the magnitude of the unit vector in each frequency bin after averaging across presentations. If the EEG waveform were completely coherent with the stimulus, and hence completely repeatable, the PLV would be 1 for each frequency bin. For random EEG responses, the angle or direction of the unit vector at each frequency would be random on each presentation, and so the magnitude of the vector after averaging tends to 0. For a given condition, epoch lists from the three sessions were first concatenated. A bootstrapping procedure was used to estimate PLVs based on 400 trials of 75 random draws from the positive and negative event lists. For each draw, a first-order Slepian window was applied before computing the Fourier transform. An average of the unit phase vectors was calculated across all draws. The PLV was averaged across all 32 channels, and peak values were extracted at key frequencies. The slow (3.3 Hz), fast (6.7 Hz), and combined (13.3 Hz) presentation rates of the tone sequences were analyzed in the low-frequency band, and the tone *F*0s (100, 106, and 238 Hz) were analyzed in the high-frequency band. A schematic showing the procedure by which the PLVs were extracted, along with a heatmap of the distribution of the PLV peak values at 3.3 Hz, is shown in Fig. 2.

## III. RESULTS

### A. Behavioral results

Participants' behavioral responses during the EEG recordings were categorized into “hit,” “false alarm,” and “random” responses. Hits were defined as button presses that occurred between 0.8 and 1.8 s after an oddball event in the target stream. False alarms were defined as button pushes that occurred between 0.8 and 1.8 s after an oddball event in the non-target stream, and random responses were all other button presses. These criteria were developed based on response timing during pilot blocks. Random responses were relatively rare, averaging less than one every other block, and so were not analyzed further.

**FIG. 3.**
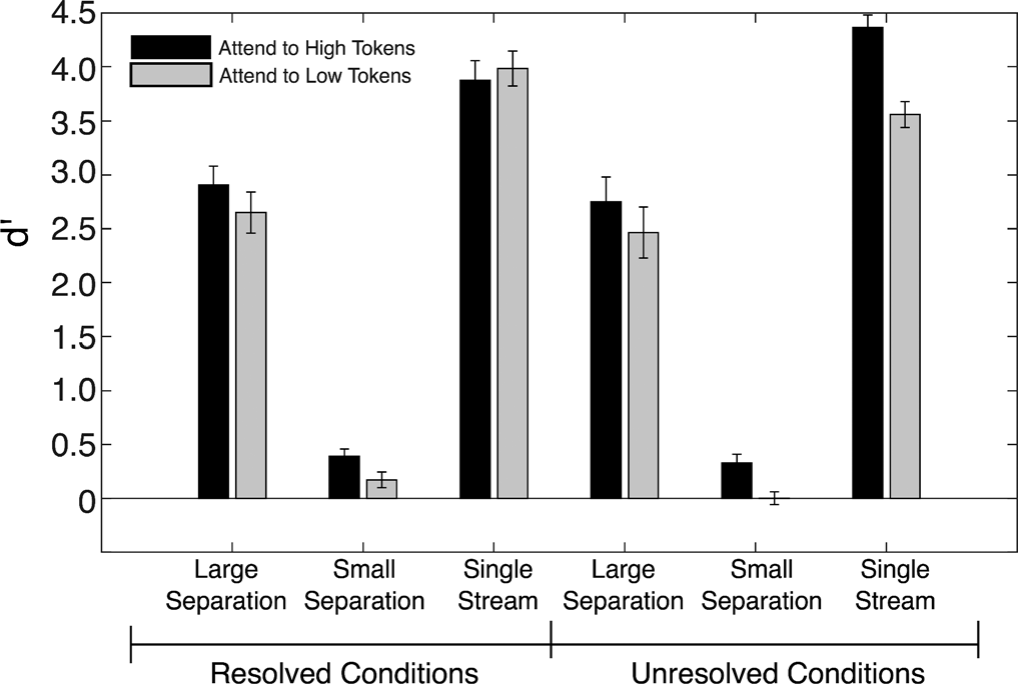
Mean behavioral performance (*N* = 19) during the EEG recordings in terms of the sensitivity index, *d′*. The results show high performance in all conditions where there is a large (15-semitone) *F*0 difference between the alternating tones, but near-chance performance when the *F*0 difference is small (one semitone). Performance was not affected by whether the harmonics were spectrally resolved. Error bars represent ±1 standard error of the mean.

Each participant's *d′* values were calculated for the 12 conditions by separately averaging hits and false alarms across the three sessions, computing the *z-*scores, and then subtracting the *z*-scored false alarms from the *z-*scored hits (Green and Swets, 1966). Participants who obtained perfect scores for a condition across all sessions were adjusted by substituting 0.995 for a perfect hit rate and 0.005 for a perfect false alarm rate, resulting in a maximum possible *d′* value of 5.15. The results shown in Fig. 3 confirm high performance for the two conditions in which only one of the two tone sequences was presented (single-stream conditions), and also show high performance in conditions with a large *F*0 separation between the A and B tones. However, when the *F*0 separation was small, performance was near chance (*d′* = 0). This pattern of results was observed for both the resolved-harmonics and unresolved-harmonics conditions.

A three-way repeated-measures analysis of variance (ANOVA) was performed on the data from the four conditions where both A and B tones were present (sequences with single tones were excluded), with *d′* as the dependent variable and factors of harmonic resolvability (resolved or unresolved), *F*0 separation (1 or 15 semitones), and attended sequence (high, A, or low, B). No main effect of resolvability was found (*F*_1,18_ = 1.5, *p* = 0.24, *η_p_*^2 ^= 0.077), confirming that the participants performed similarly with both resolved and unresolved harmonics. There was a main effect of *F*0 separation (*F*_1,18_ = 268.6, *p* < 0.0001, *η_p_*^2 ^= 0.94), confirming the observation that performance was much poorer with the 1-semitone separation than with the 15-semitone separation, in line with expectations. There was also a main effect of attended sequence (*F*_1,18_ = 9.1, *p* = 0.007, *η_p_*^2 ^= 0.34), reflecting the fact that performance was slightly poorer when listeners attended to the low tones than to the high tones. No interactions between the main effects were significant (*p* > 0.6 in all cases).

### B. EEG responses

#### 1. High-frequency responses

**FIG. 4.**
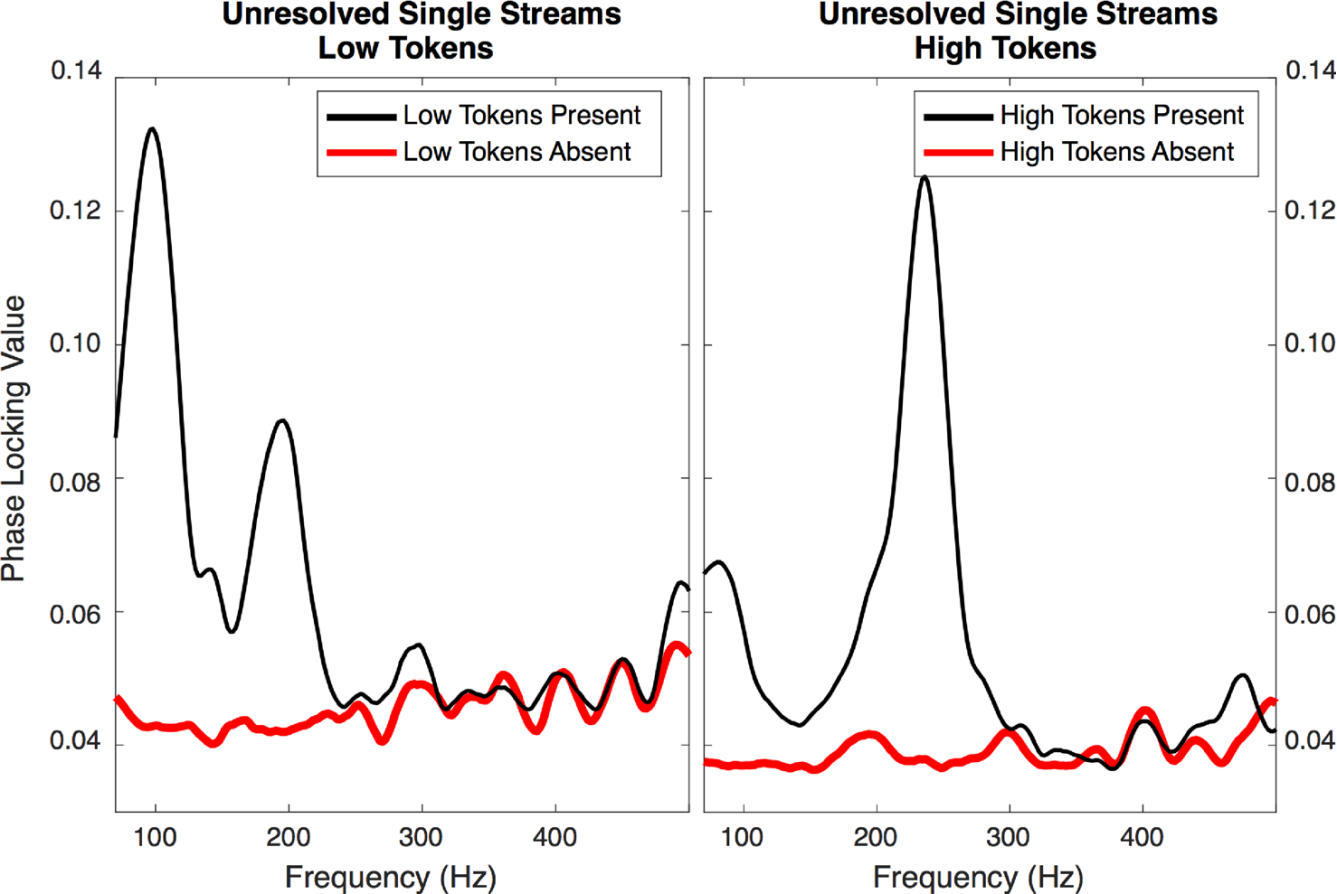
(Color online) Averaged PLV spectra of the high-frequency EFR in response to low-*F*0 (100 Hz; left panel) and high-*F*0 (238 Hz; right panel) tokens from the single-stream conditions. Thin black lines indicate conditions where the tokens were present; thicker red lines indicate where the tokens were absent (baseline). In both panels, a clear peak in the EFR is observed at the *F*0 when the stimulus was present.

The PLV spectra for the higher-frequency EEG responses demonstrate neural phase locking to the fundamental and harmonics of the tokens presented. As an example, Fig. 4 shows phase locking in the unresolved single-stream conditions averaged over all 19 participants. In these conditions, the analyzed token was present in one condition (black traces) but absent in the other (red traces).

**FIG. 5.**
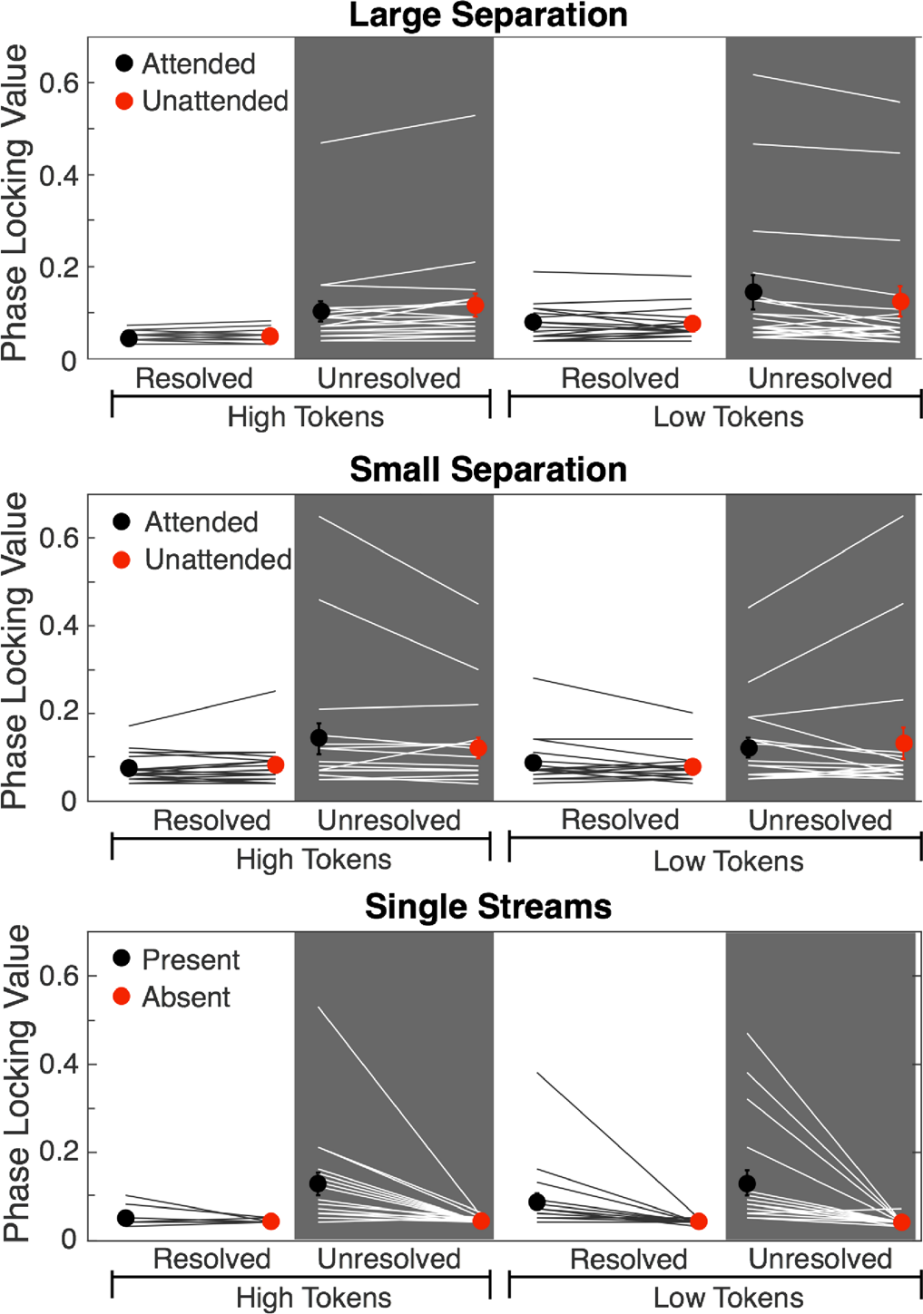
(Color online) Individual and mean PLV peak values in the high-frequency EFR for high tokens (238 Hz in Large Separation and Single Stream conditions and 106 Hz in the Small Separation condition) and for low tokens (100 Hz in all conditions). Individual data (*N* = 19) are shown as lines, and mean data are shown as symbols with error bars representing ±1 standard error of the mean. In conditions where both A and B tones are present (upper and middle panels for large and small *F*0 separations, respectively), the results show no significant effects of attention on the high-frequency EFR responses.

Token-specific phase locking to the *F*0 was also observed in conditions where the stimuli were identical (both high and low tokens present) and differentiated only by which stream the participants were instructed to attend to. In these cases, peak magnitudes were similar between conditions. Peaks were extracted from these spectra at points corresponding to the high *F*0 (238 Hz in large-separation and single-stream conditions, 106 Hz in small-separation conditions) and low *F*0 (100 Hz) in each condition; the values of these peaks are shown in Fig. 5, with the individual values as lines and the mean values as symbols.

Repeated-measures ANOVAs were conducted on the PLVs in all conditions that included both the A and B tones, separately for high and low tokens to study the main effects of separation size (1 or 15 semitones), harmonic resolvability (resolved or unresolved), and attention (attended or unattended). For the high-*F*0 tokens (A tones), there was a main effect of resolvability (*F*_1,18_ = 7.5, *p* = 0.013, *η_p_*^2 ^= 0.30), with the unresolved harmonics producing a larger PLV on average. There was also a main effect of *F*0 separation (*F*_1,18_ = 12.4, *p* = 0.002, *η*_p_^2 ^= 0.41), although it is not clear if this was due to the change in *F*0 separation between the A and B tones, or simply the different *F*0 of the A tone. There was no main effect of attention (*F*_1,18_ = 0.001, *p* = 0.982, *η_p_*^2 ^< 0.001), and no significant interactions. For the low-*F*0 tokens (B tones), the ANOVA revealed no significant main effects (Resolvability: *F*_1,18_ = 3.56, *p* = 0.075, *η_p_*^2 ^= 0.17; Separation: *F*_1,18_ = 0.59, *p* = 0.452, *η_p_*^2 ^= 0.032; Attention: *F*_1,18_ = 1.34, *p* = 0.261, *η_p_*^2 ^= 0.069), and no significant interactions (all *p* > 0.09). Thus, for the high-frequency EFR, there was no evidence for attentional modulation.

#### 2. Low-frequency responses

**FIG. 6.**
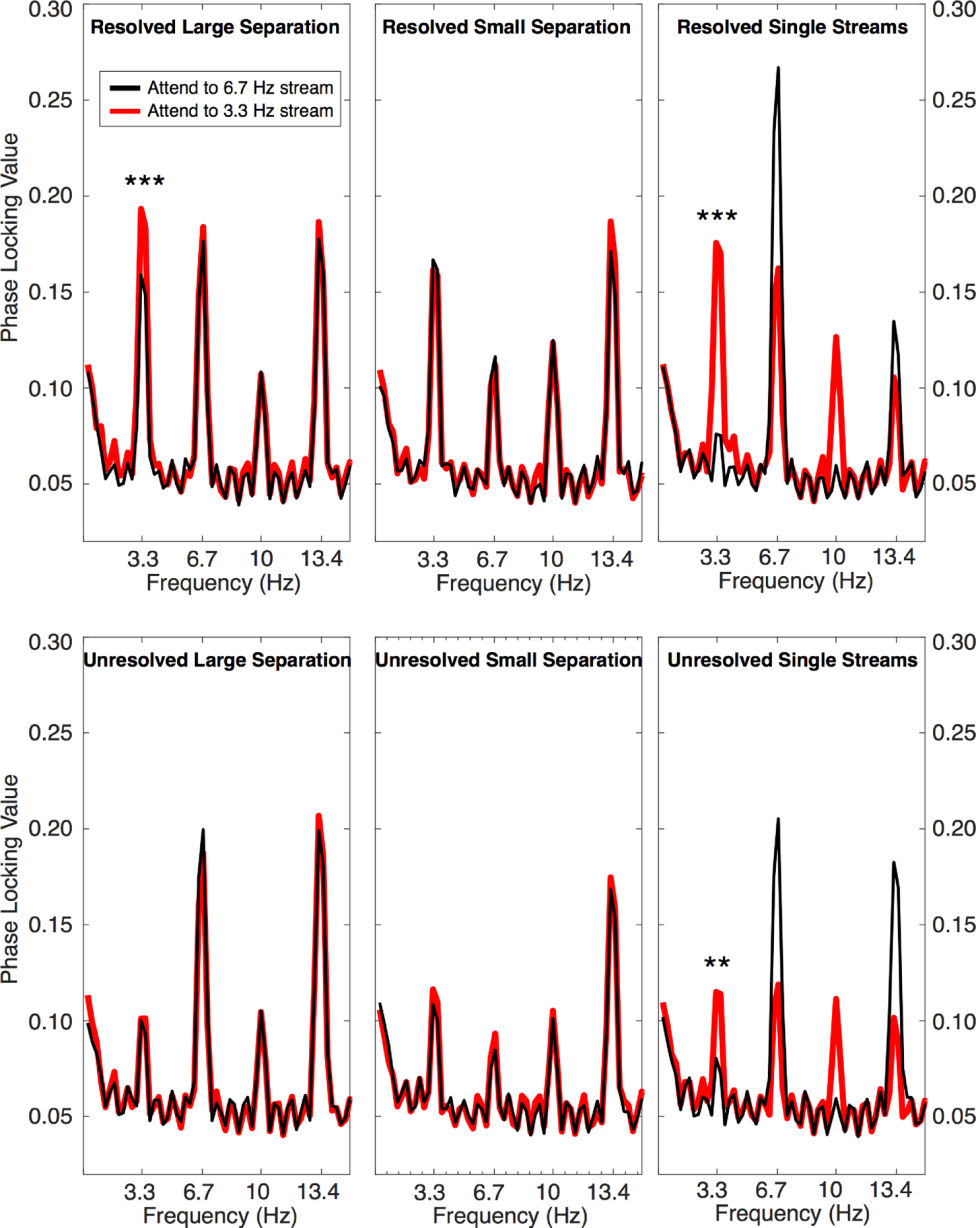
(Color online) Averaged PLVs in the range of the tone repetition rates (3.3–6.7 Hz) and their second harmonics. Upper panels show data from conditions with resolved harmonics; lower panels show data from conditions with only unresolved harmonics. Thin black lines correspond to conditions where the high, fast-rate (6.7 Hz) tones were attended; thicker red traces correspond to conditions where the low-*F*0, slow-rate (3.3 Hz) tones were attended. The results show a significant effect of attention (***p ≤ 0.001), but only in the case of the large *F*0 separation, and then only for the condition with the resolved harmonics. The far right panels show the PLV spectrum for conditions where only a single tone sequence (A-tone or B-tone) was present. For both the resolved-harmonic and unresolved-harmonic complex tones, the 3.3-Hz component was significantly larger for the B-tones alone than for the A-tones alone (**p ≤ 0.01, ***p ≤ 0.001).

The PLV spectra for the lower-frequency (cortical) band demonstrate robust neural phase locking to the presentation rates and multiples of the high (fast rate: 6.7 Hz) and/or low (slow rate: 3.3 Hz) streams in all conditions (Fig. 6). In conditions with both A and B tones with only unresolved harmonics (Fig. 6, lower left and center), there was no clear effect of attention: the red and black lines generally overlap. In contrast, in conditions with resolved harmonics and large *F*0 separation (Fig. 6, upper left panel), the amplitude of the 3.3-Hz peak seems to be modulated by attention, with its amplitude enhanced when listeners were attending to the slower-rate (3.3-Hz) low-*F*0 tones.

**FIG. 7.**
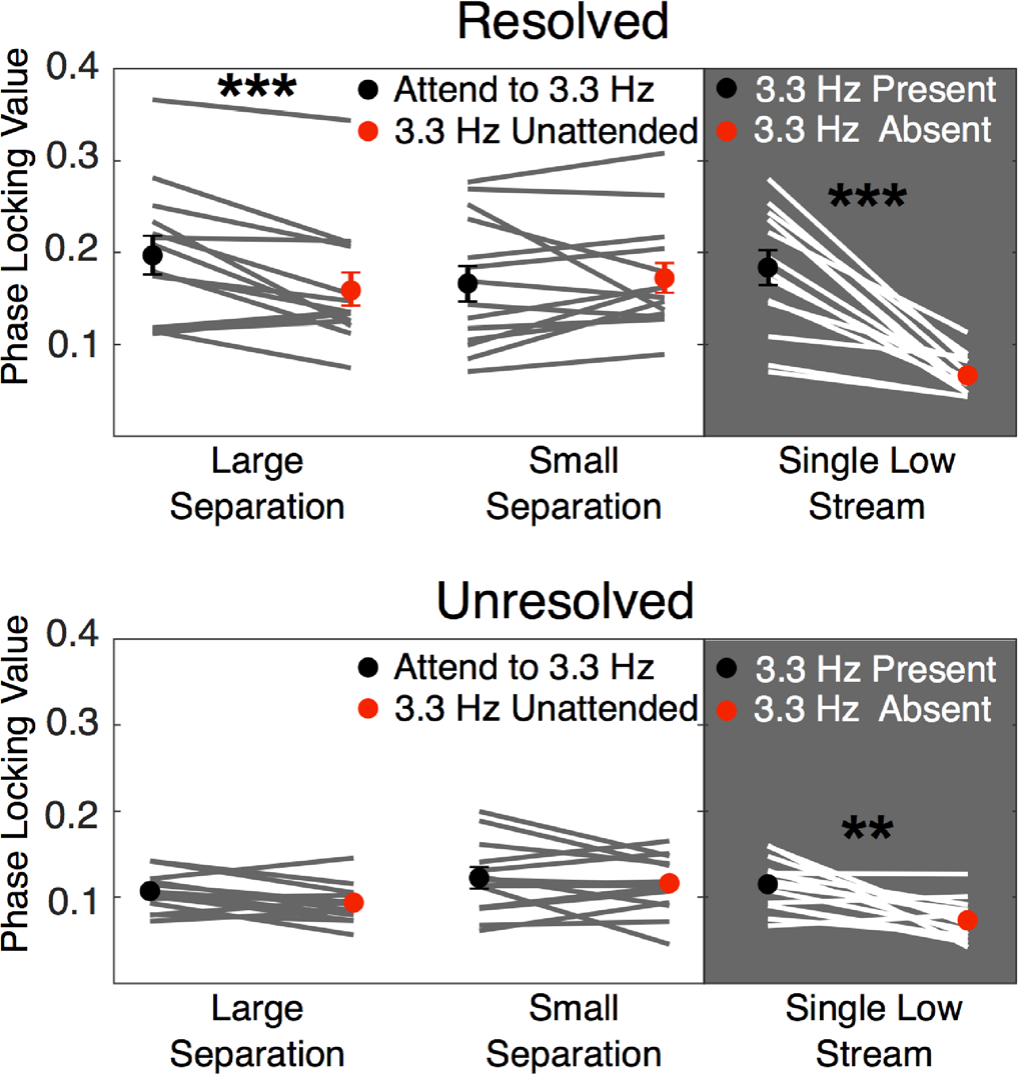
(Color online) Individual and mean PLVs at 3.3 Hz (*N* = 19). Individual data (*N* = 19) are shown as lines, and mean data are shown as symbols with error bars representing ±1 standard error of the mean. The upper panels show results from conditions with resolved harmonics; the lower panels show results from conditions with only unresolved harmonics. Far right panels show the results with only one stream present. As shown in Fig. 6, the effect of attention was significant in the two-stream conditions, but only for the condition with the large *F*0 separation, and then only in the condition with resolved harmonics (***p ≤ 0.001, **p ≤ 0.01).

Because the fast presentation rate of the A tones (6.7 Hz) is also the second harmonic of the B-tone presentation rate (3.3 Hz), the interpretation of the 6.7-Hz peak alone is problematic. We considered several methods of analyzing the data, including considering just the 3.3-Hz component, as well as calculating the ratio or difference between the PLV at 3.3 Hz and at 6.7 Hz. All approaches produced results that were highly correlated and resulted in the same statistical conclusions. The mean and individual PLVs at 3.3 Hz are shown in Fig. 7 for all the conditions tested. A three-way repeated-measures ANOVA was performed with this PLV amplitude at 3.3 Hz as the dependent variable, and factors of resolvability (resolved or unresolved), F0 separation (1 or 15 semitones), and attention (high or low tones attended). Conditions containing only one of the two tones were again excluded. A significant main effect of resolvability was found (*F*_1,18_ = 51.8, *p* < 0.0001, *η_p_*^2 ^= 0.80), but there were no main effects of either *F*0 separation (*F*_1,18_ = 0.16, *p* = 0.70, *η_p_*^2 ^= 0.012) or attention (*F*_1,18_ = 4.5, *p* = 0.053, *η_p_*^2 ^= 0.26). Two-way interactions with resolvability were not significant (resolvability × separation: *F*_1,18_ = 1.29, *p* = 0.28, *η_p_*^2 ^= 0.090; resolvability × attention: *F*_1,18_ = 0.29, *p* = 0.60, *η_p_*^2 ^= 0.022); however, there was a significant two-way interaction between *F*0 separation and attention (*F*_1,18_ = 8.03, *p* = 0.014, *η_p_*^2 ^= 0.382) and a three-way interaction (*F*_1,18_ = 9.65, *p* = 0.011, *η_p_*^2 ^= 0.40), supporting the observation that attention did affect responses, but only when the *F*0 separation was large, and then only when the harmonics were resolved. This interpretation is further supported by the fact that paired comparisons revealed a significant difference due to attention for the resolved harmonics with the large *F*0 separation (*t*_18_ = 3.9, *p* = 0.001), but no difference due to attention for the resolved harmonics with the small *F*0 separation (*t*_18_ = 0.55, *p* = 0.59), or for the unresolved harmonics with either the large *F*0 separation (*t*_18_ = 0.13, *p* = 0.9) or small *F*0 separation (*t*_18_ = 1.1, *p* = 0.27). Overall, therefore, attention affected only the low-frequency (cortical) responses, and then only in the condition that induced stream segregation with spectrally resolved harmonics.

## IV. DISCUSSION

### A. Behavioral outcomes confirm perceptual streaming in the absence of tonotopic cues

The behavioral results show that listeners are able to detect oddballs accurately in one of the tone streams when the tone streams are presented in isolation, and when there is a large *F*0 difference between the attended and unattended stream. High levels of performance were observed regardless of whether the harmonics in the complex tones were spectrally resolved or not. This outcome confirms that listeners are able to segregate sequences into streams based on *F*0 differences, even when the harmonics are all unresolved, resulting in no tonotopic cues (Vliegen and Oxenham, 1999; Grimault *et al.*, 2000).

### B. High-frequency EFRs show no attention-based streaming effects

The EFR to high frequencies ( >80 Hz) has until recently been considered to primarily reflect subcortical activity from brainstem and midbrain nuclei, in particular the inferior colliculus (Kiren *et al.*, 1994; Kuwada *et al.*, 2002; Shinn-Cunningham *et al.*, 2017). Some recent work using magnetoencephalography (MEG) and EEG has suggested that responses up to around 100 Hz may also include cortical generators (Coffey *et al.*, 2016; Coffey *et al.*, 2017); however, the most recent work on the topic suggests that with EEG (as opposed to MEG), responses around 100 Hz remain strongly dominated by subcortical structures (Bidelman, 2018).

Some earlier studies have suggested that neural correlates of streaming may emerge in subcortical structures (Pressnitzer *et al.*, 2008; Schadwinkel and Gutschalk, 2011; Yao *et al.*, 2015), and one study has reported modulation of the subcortical frequency following response (FFR) by perceived streaming (whether one or two streams were perceived in a repeating ABA triplet sequence) (Yamagishi *et al.*, 2016). However, in that study, the effect was only observed for the second A tone of each triplet, whereas cortical correlates have tended to be observed for the B tone in each sequence (Gutschalk *et al.*, 2005; Yamagishi *et al.*, 2016). In general, reports of attentional effects on subcortical responses have been rare (e.g., Galbraith *et al.*, 1998; Hairston *et al.*, 2013), and a review of the earlier literature concluded that there was little or no evidence supporting the attentional modulation of the EFR (Varghese *et al.*, 2015). Most recently, a study found attentional modulation of the EFR to frequencies less than 100 Hz, but not to frequencies around 200 Hz (Holmes *et al.*, 2017).

Our results using *F*0s between 100 and 231 Hz showed a strong response to the *F*0 of tones in the sequence and its harmonics. In general, the PLVs in response to tones containing only unresolved harmonics were larger than the PLVs in response to tones containing resolved harmonics. Thus, the change in PLV amplitude from resolved to unresolved harmonics is opposite to the change in perceptual pitch strength or accuracy found in many previous studies (Houtsma and Smurzynski, 1990; Shackleton and Carlyon, 1994; Bernstein and Oxenham, 2003). The dissociation between PLV and pitch strength is consistent with the idea that the EFR reflects stimulus periodicity but not the perception of pitch (Gockel *et al.*, 2011). Despite the robust high-frequency EFRs in our study, no effects of attention were observed in any condition. Thus, our results provide no evidence for the emergence of auditory stream-based attentional effects in subcortical structures.

### C. Low-frequency EFRs reflect tonotopic-based attentional streaming

A different pattern of results was observed in the lower-frequency EEG band, phase-locked to the stimulus repetition rates, which likely reflects cortical activity (Kuwada *et al.*, 2002). In contrast to the high-frequency EFR, a robust effect of attention was observed here, but only in the case of the large *F*0 separation with the spectrally resolved harmonics. The lack of an attentional effect with the small *F*0 separations is consistent with the behavioral results: the *F*0 difference was too small to induce sequential streaming between the two alternating tones, resulting in near-chance performance in the behavioral task, which in turn indicated an inability to attend selectively to one or other sequence. However, the lack of an attentional effect on the cortical PLV with the unresolved harmonics and large *F*0 separation represents a dissociation between the cortical and the behavioral results. It therefore appears that the EEG correlates of streaming and attention in our paradigm are limited to conditions where there are tonotopic differences between the A- and B-tone sequences.

As mentioned in the Introduction, there have been numerous reports of attentional modulation of auditory cortical responses in the past, using EEG (e.g., Hillyard *et al.*, 1973), MEG (Gutschalk *et al.*, 2008; Xiang *et al.*, 2010), functional magnetic resonance imaging (fMRI) (Petkov *et al.*, 2004), and electrocorticography (ECoG) (Mesgarani and Chang, 2012). Neural correlates of streaming for pure tones have been reported previously in humans (Cusack, 2005; Gutschalk *et al.*, 2005; Snyder *et al.*, 2006; Wilson *et al.*, 2007) and other species (Fishman *et al.*, 2001; Micheyl *et al.*, 2005; Bee *et al.*, 2010; Itatani and Klump, 2014). Neural correlates of streaming have also been reported based on higher-level features, such as pitch in humans (Gutschalk *et al.*, 2007) and spatial separation in both humans (Schadwinkel and Gutschalk, 2010) and other species (Yao *et al.*, 2015). However, none of these studies on higher-level features explicitly examined the role or correlates of attention in their tasks. Thus, to our knowledge, this is the first study to examine the correlations of attention to specific sound dimensions in a streaming paradigm. Our results suggest that the tonotopic, but not the periodicity, dimension produces measurable attention-based modulation of the cortical EFRs.

## V. CONCLUSIONS

Despite strong responses to the stimuli, no attentional modulation of high-frequency EFRs was observed, consistent with no modulation of phase-locked brainstem or midbrain responses. Low-frequency EFR provided correlates of attention to streams that are likely to be cortical in nature, but only in conditions where some tonotopic differences existed between the alternating tones in the sequences. Our findings should not be interpreted as evidence against a neural representation of non-tonotopic auditory streaming; instead, it may be that the population-based EEG recordings are not sensitive to the potentially smaller neural populations that respond selectively to higher-level features, such as pitch. As an example, a search for pitch-sensitive neurons in the auditory cortex of marmosets found only a relatively small number of such units in a relatively constrained region of auditory cortex (Bendor and Wang, 2005), whereas large areas of primary auditory cortex are known to reflect tonotopic organization in response to both simple (Formisano *et al.*, 2003) and complex (Moerel *et al.*, 2012) sounds. Taken together with earlier findings, our results suggest that EEG measures can provide correlates of auditory streaming and attention, but that such population-based correlates may be sensitive primarily to low-level tonotopic differences, and not to higher-level features, such as pitch, that extend beyond simple tonotopy.
